# Supplementary material for: Cloud BioLinux: pre-configured and on-demand bioinformatics computing for the genomics community
Source: BMC Bioinformatics. 2012 Mar 19;13:42. doi: 10.1186/1471-2105-13-42 (PMC3372431; doi:10.1186/1471-2105-13-42)
Supplement: Additional file 1 — Supplementary 1 Cloud BioLinux software documentation in the form of a mini, self-contained website. Users need to download and uncompress the .zip file, and open through a web browser the "index.html" file available on the main directory. (ZIP 1823 kb). [file 1471-2105-13-42-S1.ZIP › Cloud-BioLinux-Package-Documentation/docs/extract.html]

Bio-Linux Software Documentation Pages

Back to search form

## extract

|  |  |
| --- | --- |
| Name | extract |
| Description | **extract** is a part of the Glimmer package, for finding genes in microbial DNA, especially the genomes of bacteria, archaea, and viruses.  The program **extract** takes a FASTA format sequence file and a file with a list of start/stop positions in that file (e.g., as produced by the long-orfs program) and extracts and outputs the specified sequences.  The first command-line argument is the name of the sequence file, which must be in FASTA format.  The second command-line argument is the name of the coordinate file. It must contain a list of pairs of positions in the first file, one per line. The format of each entry is:  `"IDstring" "start position" "stop position"` (where "" is replaced by open and close arrows).  This file should contain no other information, so if you're using the output of glimmer or long-orfs , you'll have to cut off header lines.  The output of the program goes to the standard output and has one line for each line in the coordinate file. Each line contains the IDstring , followed by white space, followed by the substring of the sequence file specified by the coordinate pair. Specifically, the substring starts at the first position of the pair and ends at the second position (inclusive). If the first position is bigger than the second, then the DNA reverse complement of each position is generated. Start/stop pairs that "wrap around" the end of the genome are allowed.  There are two optional command-line arguments:  - -skip makes the output omit the first 3 characters of each sequence, i.e., it skips over the start codon. This was the default behaviour of the previous version of the program. - -l n makes the output omit an sequences shorter than n characters. n includes the 3 skipped characters if the -skip switch is one.  **References:**  Salzberg SL, Delcher AL, Kasif S, White O: Microbial gene identification using interpolated Markov models. Nucleic Acids Res. 1998 Jan 15;26(2):544-8. [Entrez]    Delcher AL, Harmon D, Kasif S, White O, Salzberg SL: Improved microbial gene identification with GLIMMER. Nucleic Acids Res. 1999 Dec 1;27(23):4636-41. [Entrez] |
| Homepage | http://www.tigr.org/software/glimmer/ |
| Remote Documentation | http://www.tigr.org/software/glimmer/glimmer.readme |
